# Does the charitable medical assistance program impact catastrophic medical expenditures for families of children with leukemia? An evidence-based study in China

**DOI:** 10.1186/s12939-025-02442-1

**Published:** 2025-03-17

**Authors:** Jun Su, Yu-qing Zhang, Di Shao, Jia-min Wang, Wei Hao, Yan-xiu Liu, Long Wang, Xiao-jie Sun

**Affiliations:** 1 Department of Social Medicine and Health Management, School of Public Health, Cheeloo College of Medicine, Shandong University, Jinan, Shandong 250012 China; 2 NHC Key Laboratory of Health Economics and Policy Research (Shandong University), Jinan, Shandong 250012 China; 3 Center for Health Management and Policy Research, Shandong University (Shandong Provincial Key New Think Tank), Jinan, Shandong 250012 China

**Keywords:** Pediatric leukemia, Catastrophic health expenditure, Charitable medical assistance, Healthcare security

## Abstract

**Background:**

Pediatric leukemia is the most prevalent childhood cancer in China, exerting a considerable financial impact on affected families. Despite the mandatory participation of all Chinese children in the Resident Basic Medical Insurance, out-of-pocket (OOP) expenses remain substantial for families of children with leukemia. However, charity assistance has been shown to help mitigate these financial burdens. The “Love Union Project” is a comprehensive charitable medical assistance program designed to support families of children with leukemia within China’s multi-tiered healthcare security system. This study was designed to evaluate the impact of the “Love Union Project” on reducing the incidence of catastrophic health expenditure (CHE) among families of children with leukemia in China.

**Methods:**

The study involved 85 children in the intervention group from H city and 36 matched control children from S and Y cities. Data on demographics, medical expenses, and assistance were collected. Non-normally distributed costs were reported as medians. Multivariate logistic regression analyzed the impact of the “Love Union Project” on CHE.

**Results:**

Thanks to the program’s intervention, the CHE rate among the intervention group decreased from 75.3% to 65.9%, while the incidence of CHE in the control group was 75.0%. Compared to families with children aged 0–6 years, those aged 7–12 were more likely to incur CHE (OR 5.224; 95% confidence intervals 1.412–19.322). Families with five or more members were also at higher risk of CHE than those with four members or fewer (OR 2.847; 95% confidence intervals 1.056–7.676). Additionally, families with a monthly income of CNY8000($1,120) or more were less likely to experience CHE than those with a monthly income of CNY4000($560) or less (OR 0.257; 95% confidence intervals 0.072–0.923). Lastly, families receiving assistance from the “Love Union Project” reported significantly lower CHE rates than those who didn’t receive such support (OR 0.151; 95% confidence intervals 0.044–0.524).

**Conclusion:**

While medical insurance provides limited relief, the “Love Union Project” enhances economic resilience for families of children with leukemia. Attention should focus on younger patients, larger households, lower-income families, and those not receiving charity support.

**Supplementary Information:**

The online version contains supplementary material available at 10.1186/s12939-025-02442-1.

## Introduction

Pediatric cancer, particularly leukemia, is a significant health challenge, being the second leading cause of death among children and adolescents aged 0 to 19 [1, 2]. In China, leukemia accounts for 48.1% of pediatric oncology discharges and 32.9% of new cases from 2019 to 2020 [3]. Treatment costs for leukemia can range from CNY 300,000 ($41,512) to over CNY 1,000,000 ($138,373) for transplants [4, 5]. This financial burden often forces families to forgo or discontinue necessary treatments, creating a substantial barrier to access for affected children [6–8]. Despite advancements in medical technology improving survival rates, the economic strain remains a critical issue for families and society [9].

To enhance treatment and support for children with leukemia in China, various governmental policies and regulations have been introduced [10]. In 2019, the State Council of China mandated the integration of basic medical insurance, critical illness insurance, and medical assistance to alleviate the financial burden on pediatric leukemia patients. In 2020, the Nanchang Municipal Government advocated for the inclusion of these patients in regular management and provision of free treatment. Additionally, public welfare charity foundations have increasingly focused on supporting children with severe illnesses. The “Love Union Project” targets pediatric leukemia in Heyuan, Guangdong Province, by providing supplementary reimbursement for medical expenses beyond basic medical insurance and critical illness coverage. This comprehensive assistance model encompasses cost reimbursement, cross-region medical treatment support, family care, and caregiver guidance, significantly mitigating the financial challenges faced by affected families.

Existed research has extensively explored the benefits of charitable medical assistance, highlighting its role in alleviating household financial stress in countries such as Jordan and Egypt [11, 12]. Studies focusing on families of children with leukemia indicated positive effects from various perspectives, including the evolution of medical security and the contributions of major illness social assistance platforms [4, 13, 14]. However, some scholars argued that this assistance may not significantly alleviate the economic burden faced by recipient families [15, 16]. Moreover, the literature revealed a lack of consensus regarding the combined effects of charitable assistance and medical insurance, with many studies lacking control groups to adequately evaluate these impacts.

Catastrophic health expenditure (CHE) is a significant indicator for measuring the economic burden of diseases and the equity of health care [17]. The study aimed to identify contributing factors, and evaluate the effectiveness of the “Love Union Project” in reducing CHE. Findings will support governmental efforts to enhance the medical insurance system for pediatric leukemia and promote the development of a multi-tiered medical security system.

## Methods

### Study design

**Fig. 1:**
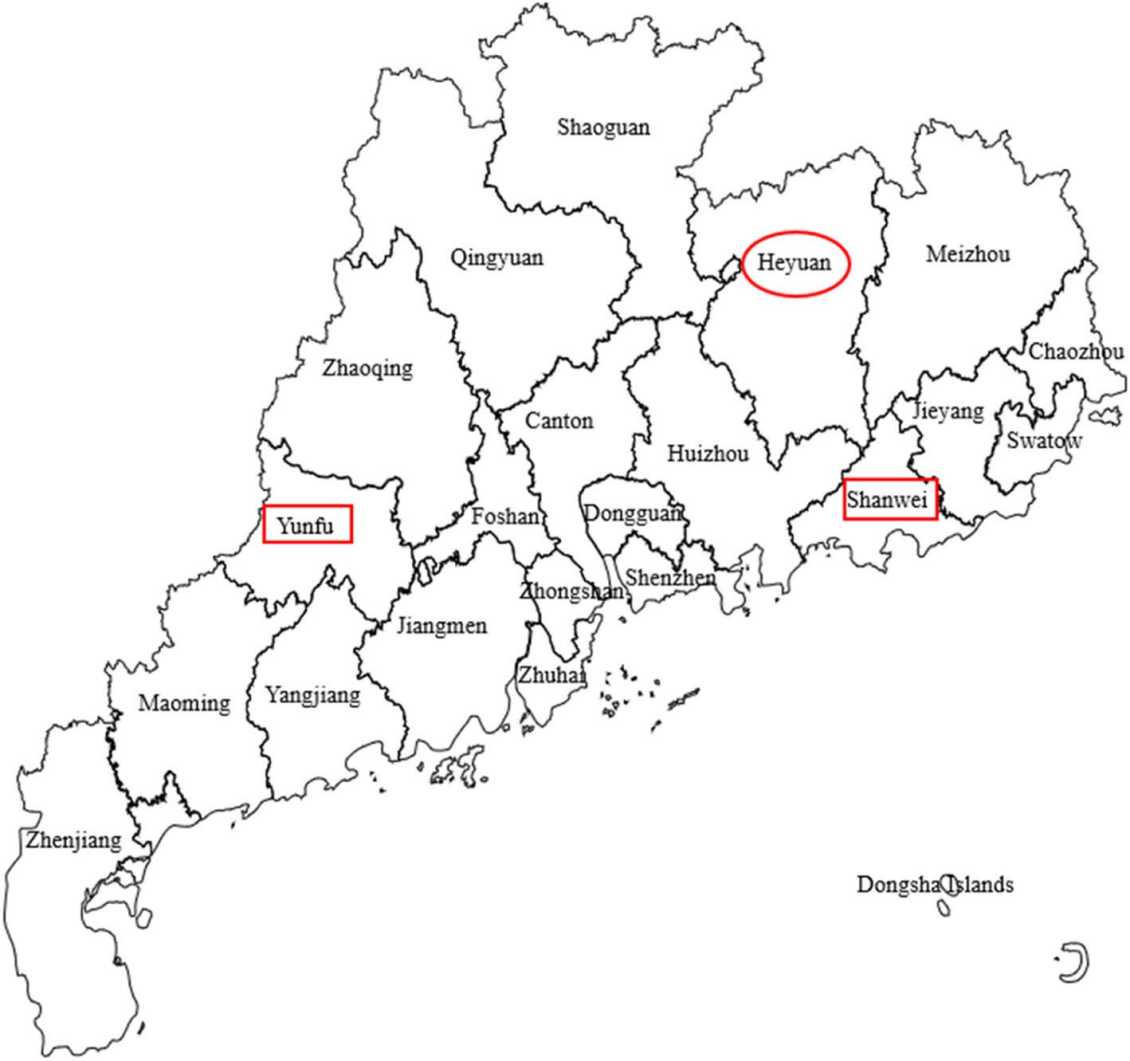
Geographical distribution of the sample

For this study, a cluster sampling method was utilized to select subjects for the intervention group, with the following inclusion criteria: (1) participation in the “Love Union Project” from August 15, 2017 to August 15, 2022; (2) the child’s registered residence is in Heyuan (Fig. 1); (3) the child is under 18 years of age (excluding those who are exactly 18 years old); (4) the child is in the maintenance period or the consolidation period. A total of 85 subjects were ultimately included in the study.

This study selected Shanwei City and Yunfu City in Guangdong Province as the control sample areas, based on similar geographical distribution, socio-economic development, healthcare advancement level, comparable medical insurance policies, and recommendations from senior local experts in pediatric leukemia treatment. A snowball sampling method was used to identify subjects for the control group, with the following inclusion criteria: (1) the child is under 18 years of age (excluding those who are exactly 18 years old); (2) the child’s household registration is in Shanwei or Yunfu (Fig. 1); (3) the location of the child’s basic medical insurance matches the household registration location; (4) the child is in the maintenance period or the consolidation period. This study invited 60 caregivers of pediatric leukemia through three avenues: a registration form on the Questionnaire Star Platform (this is a professional online survey platform that provides a series of services including questionnaire design, data collection, and analysis of survey results), mutual referrals among caregivers, and medical staff referrals. After excluding 16 cases not meeting the inclusion criteria and 8 cases refusing to participate, a total of 36 caregivers provided informed consent forms and successfully completed the survey.

### Measurements

Between June and August 2022, an online survey was conducted among the two groups of eligible pediatric leukemia patients and their caregivers. The survey covered demographic characteristics (such as age, gender, family size, monthly household income), clinical characteristics (including disease duration, disease type, severity of illness, treatment stage, infection status, transplantation status, and comorbid conditions), inpatient out-of-pocket expenses, costs for medications purchased outside the hospital, other outpatient expenses, and the reimbursement amounts from basic medical insurance and critical illness insurance. Additionally, project financial assistance details were provided by the staff of the “Love Union Project”.

This study employed Out-Of-Pocket (OOP) expenses and the incidence of CHE to reflect the financial burden on families of children with leukemia. OOP includes inpatient self-pay expenses, outpatient medication costs, and other outpatient self-pay expenses. The study adopted the World Health Organization’s definition of CHE, which is considered to occur when the ratio of a family’s OOP health care costs to its capacity to pay reaches or exceeds 40% [18].

### Statistical analysis

Epi Data 3.1 software was utilized to establish the database, and Excel 2019 and SPSS 26.0 were employed for the statistical analysis. Descriptive analysis was used for the basic characteristics of the pediatric patients. Normal distribution continuous variables were described by the mean ± standard deviation ($\overline{x }\pm s$), whereas non-normally distributed continuous variables were characterized by the median. Categorical variables were expressed as frequencies and percentages (%), and Chi-square tests were used for group comparisons. Cost data were represented by the median, with the Wilcoxon rank-sum test applied for group comparisons. Chi-square test was used for univariate analysis, and multivariate Logistic regression analysis was conducted for identifying influencing factors. Two-tailed *P* < 0.05 was considered statistically significant.

### Ethical approval

The research adhered to the ethical guidelines of the Declaration of Helsinki. Approval was granted by the Medical Ethics Committee of the Center for Health Management and Policy Research at Shandong University (No. ECSHCMSDU 20211101). Before starting the survey, the researchers explained its goals and details to the child’s caregivers and began only after receiving their informed consent. During the survey, caregivers had the right to withdraw at any time. The information of the pediatric patients and their caregivers in this study will be strictly confidential and used only for academic purposes.

## Results

### Demographic and clinical characteristics

In the intervention group, the mean age of the 85 pediatric patients was 9.96 ± 3.76 years. The majority of the patients were male (62.4%), predominantly with Acute Lymphocytic Leukemia (81.2%), with moderate disease risk (49.4%), in the consolidation period (55.3%), and from non-poverty households (78.8%).

In the control group, the mean age of the 36 pediatric patients was 8.89 ± 3.99 years. The majority of the patients were male (61.1%), predominantly with Acute Lymphocytic Leukemia (83.3%), with moderate disease risk (41.7%), in the maintenance period (77.8%), and from non-poverty households (61.1%).

Chi-squared tests revealed a statistically significant difference in the treatment phases between the two groups (*P* < 0.05). The consolidation period was more frequently observed in the intervention group than in the control group, as detailed in eTable 1.

### The incidence of CHE among families of children with leukemia

**Fig. 2:**
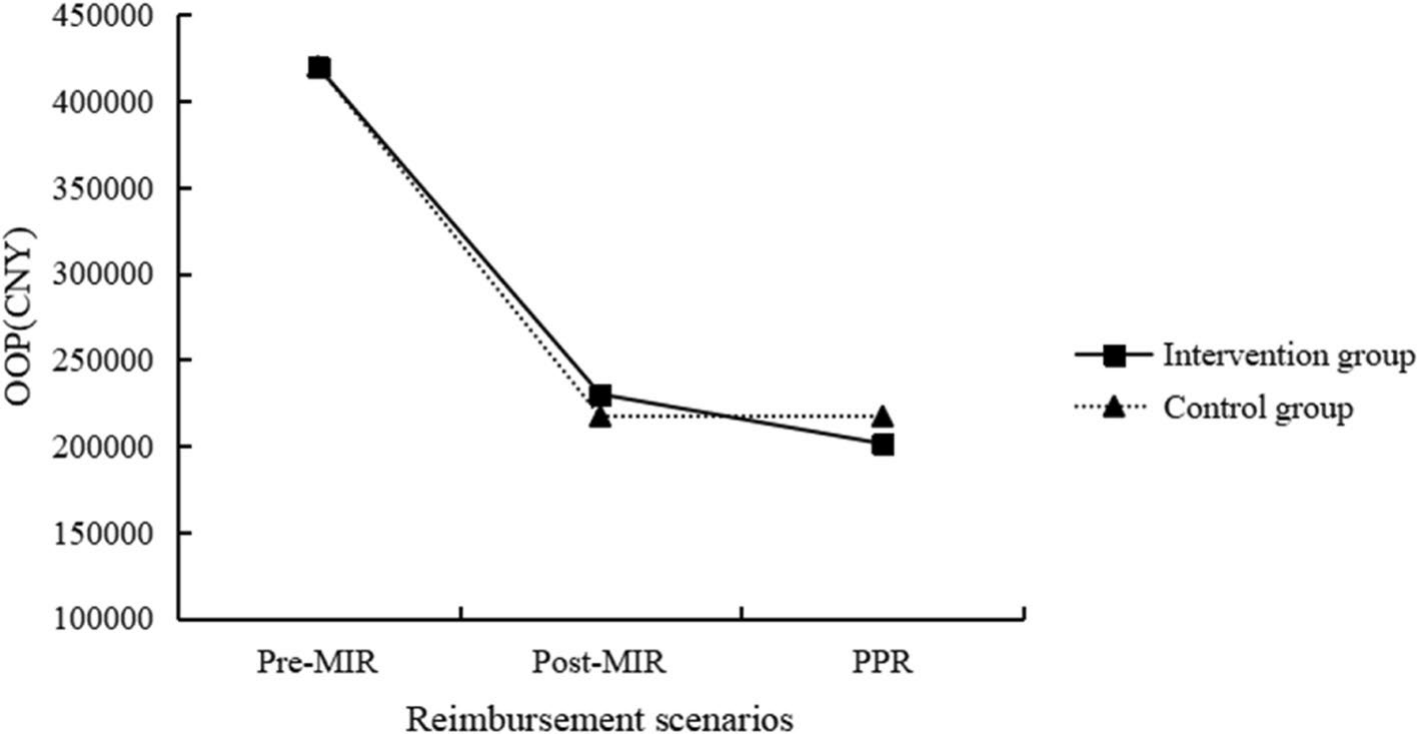
Intervention and control groups’ OOP expenses across three reimbursement scenarios. (Note: OOP, Out-of-Pocket; MIR, Medical Insurance Reimbursement; CNY, Chinese Yuan; PPR, Post-Project reimbursement, the scenario was conducted on the intervention group with the project aid and compared with the control group after medical insurance reimbursement)

**Fig. 3:**
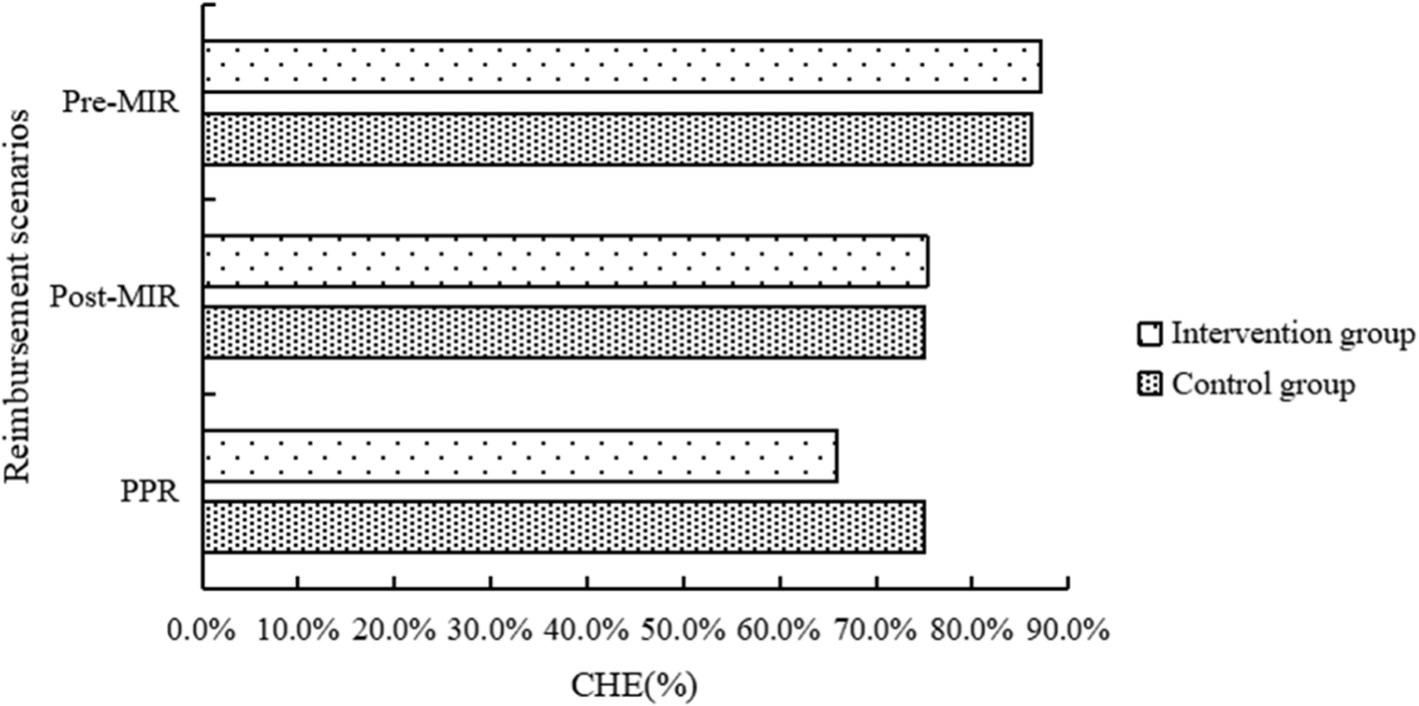
Incidence of CHE in intervention and control groups across three reimbursement scenarios. (Note: OOP, Out-of-Pocket; MIR, Medical Insurance Reimbursement; CNY, Chinese Yuan; PPR, Post-Project reimbursement, the scenario was conducted on the intervention group with the project aid and compared with the control group after medical insurance reimbursement)

Figures 2 and 3 displayed the OOP expenses and CHE among the families of children with leukemia in two groups under three reimbursement scenarios. After medical insurance reimbursement, the OOP payments for the intervention group and the control group were reduced to CNY230,000($31,825) and CNY217,200($30,054), with a decrease of 45.2% and 48.3%, respectively. Following additional charitable medical assistance reimbursement, the OOP payment for the intervention group further decreased by 12.5% to CNY201,300($27,854). Wilcoxon rank-sum test revealed no statistically significant differences in OOP expenses between the two groups across the three scenarios (*P* > 0.05) (Table 1). After medical insurance reimbursement, the incidence of CHE for the intervention and control groups decreased to 75.3% and 75.0%, with a reduction of 13.5% and 12.9%, respectively. After further supplementary reimbursement from the program, the incidence of CHE for the intervention group decreased by an additional 12.5%. Chi-squared tests showed no statistically significant difference in the incidence of CHE between the two groups (*P* > 0.05) (Table 2). Subgroup analysis indicated that there were no statistically significant differences in OOP payments and the incidence of CHE among families with children in the maintenance period or consolidation period across the three different reimbursement scenarios (*P* > 0.05), as shown in eTables 2 to 5.

**Table 1:** Comparison of OOP in families of children with leukemia across different scenarios

Scenarios	Intervention group(*n* = 85)		Control group(*n* = 36)		*Z*	*P*
	Median	Reduction (%)	Median	Reduction (%)		
Pre-MIR	CNY420,000	/	CNY420,100	/	−0.128	0.898
Post-MIR	CNY230,000	45.2	CNY217,200	48.3	−0.845	0.398
PPR	CNY201,300	12.5	/	/	−0.471	0.638

*OOP* Out-of-Pocket, *MIR* Medical Insurance Reimbursement, *CNY* Chinese Yuan, *PPR* Post-Project reimbursement, the scenario was conducted on the intervention group with the project aid and compared with the control group after medical insurance reimbursement

**Table 2:** Comparison of CHE incidence across different scenarios in families of children with leukemia

Scenarios	Intervention group(*n* = 85)		Control group(*n* = 36)		*χ*^*2*^	*P*
	CHE, n (%)	Reduction (%)	CHE, n (%)	Reduction (%)		
Pre-MIR	74(87.1)	/	31(86.1)	/	< 0.001	0.898
Post-MIR	64(75.3)	13.5	27(75.0)	12.9	0.001	0.398
PPR	56(65.9)	12.5	/	/	0.976	0.638

*CHE* Catastrophic Health Expenditure, *MIR* Medical Insurance Reimbursement, *PPR* Post-Project reimbursement, the scenario was conducted on the intervention group with the project aid and compared with the control group after medical insurance reimbursement

### Determinants of CHE in pediatric families

The univariate analysis showed that the receipt of charitable medical assistance was a significant determinant factor for the incidence of CHE in families of children with leukemia (*χ*^*2*^ = 4.134, *P* = 0.042) (see Table 3). Based on literature review and the univariate analysis, a multivariate Logistic regression analysis was conducted with variables potentially affecting the incidence of CHE as independent variables and the incidence of CHE as the dependent variable. Collinearity diagnostic analysis, with variance inflation factors for the independent variables between 1.066 and 1.454, confirmed the absence of multicollinearity. The results indicated that the model fit was satisfactory (*P* = 0.652). The probability of the incidence of CHE was lower in families with a monthly income above CNY8,000 (*OR* = 0.257, *95% CI:* 0.072–0.923) and those receiving charitable medical assistance (*OR* = 0.151, *95% CI:* 0.044–0.524). Conversely, the probability was higher for families with children aged 7–12 years (*OR* = 5.224, *95% CI:* 1.412–19.322) and family sizes of five or more (*OR* = 2.847, 95% *CI:* 1.056–7.676), as shown in Table 4.

**Table 3:** The univariate analysis of the incidence of CHE for families of children with leukemia(*N* = 121)

Variables	CHE		*χ*^*2*^	*P*
	With(*n* = 74)	Without(*n* = 47)		
Sex, n (%)			0.003	0.959
Male	46(61.3)	29(38.7)		
Female	28(60.9)	18(39.1)		
Age, n (%)			0.889	0.641
0–6 years	15(55.6)	12(44.4)		
7–12 years	41(65.1)	22(34.9)		
13–18 years	18(58.1)	13(41.9)		
Disease type, n (%)			0.048	0.826
ALL	61(61.6)	38(38.4)		
Other disease types	13(59.1)	9(40.9)		
Severity of illness, n (%)			1.706	0.426
Low risk	16(51.6)	15(48.4)		
Moderate risk	36(63.2)	21(36.8)		
High risk	22(66.7)	11(33.3)		
Treatment stage, n (%)			0.057	0.812
Maintenance period	41(62.1)	25(37.9)		
Consolidation period	33(60.0)	22(40.0)		
Transplant status, n (%)			0.005	0.943
Yes	31(60.8)	20(39.2)		
No	43(61.4)	27(38.6)		
Infection status, n (%)			1.010	0.315
Yes	40(65.6)	21(34.4)		
No	34(56.7)	26(43.3)		
Comorbidities, n (%)			0.001	0.976
Yes	8(61.5)	5(38.5)		
No	66(61.1)	42(38.9)		
Time since diagnosis, n (%)			1.242	0.265
0–24 months	19(70.4)	8(29.6)		
> 24 months	55(58.5)	39(41.5)		
Frequency of hospital visits, n (%)			1.643	0.440
1–2 times monthly	47(58.0)	34(42.0)		
3–4 times monthly	12(75.0)	4(25.0)		
≥ 5 times monthly	15(62.5)	9(37.5)		
Household size, n (%)			2.157	0.142
1–4 members	19(51.4)	18(48.7)		
5 or more	55(65.5)	29(34.5)		
One-Child family, n (%)			< 0.001	1.000
Yes	6(66.7)	3(33.3)		
No	68(60.7)	44(39.3)		
Monthly family income, n (%)			4.798	0.187
[0 ~ 4000] CNY	24(75.0)	8(25.0)		
(4000 ~ 5500] CNY	17(58.6)	12(41.4)		
(5500 ~ 8000] CNY	18(62.1)	11(37.9)		
(8000 ~ + ∞) CNY	15(48.4)	16(51.6)		
Charitable medical assistance, n (%)			4.134	0.042^*^
Yes	47(55.3)	38(44.7)		
No	27(75.0)	9(25.0)		

*CHE* Catastrophic Health Expenditure, *ALL* Acute Lymphocytic Leukemia, *CNY* Chinese Yuan

^*^P < 0.05

**Table 4:** Multivariate logistic regression analysis of CHE in pediatric leukemia families(*N* = 121)

Independent variables	*B*	*S.E*	*Wald*	*P*	*OR (95%CI)*
Age (Ref. 0–6 years)					
7–12 years	1.653	0.667	6.136	0.013^*^	5.224(1.412–19.322)
13–18 years	0.867	0.695	1.557	0.212	2.380(0.610–9.294)
Severity of illness (Ref. Low risk)					
Moderate risk	1.064	0.612	3.025	0.082	2.898(0.874–9.613)
High risk	1.167	0.713	2.679	0.102	3.214(0.794–13.005)
Treatment stage (Ref. Consolidation period)					
Maintenance period	−0.454	0.530	0.733	0.392	0.635(0.225–1.795)
Comorbidities (Ref. No)					
Yes	0.528	0.725	0.530	0.467	1.695(0.409–7.021)
Transplant status (Ref. No)					
Yes	−0.414	0.471	0.770	0.380	0.661(0.262–1.666)
Infection status (Ref. No)					
Yes	0.482	0.481	1.005	0.316	1.620(0.631–4.157)
Time since diagnosis (Ref. 0–24 months)					
> 24 months	−1.211	0.669	3.276	0.070	0.298(0.080–1.106)
Frequency of hospital (Ref. 1–2 times monthly)					
3–4 times monthly	0.602	0.752	0.640	0.424	1.826(0.418–7.978)
≥ 5 times monthly	−0.450	0.590	0.582	0.446	0.637(0.200–2.027)
Household size (Ref. 1–4 members)					
5 or more	1.046	0.506	4.276	0.039^*^	2.847(1.056–7.676)
Monthly family income (Ref. [0 ~ 4000] CNY)					
(4000 ~ 5500] CNY	−1.221	0.674	3.283	0.070	0.295(0.079–1.105)
(5500 ~ 8000] CNY	−0.406	0.652	0.387	0.534	0.666(0.186–2.392)
(8000 ~ + ∞) CNY	−1.358	0.652	4.341	0.037^*^	0.257(0.072–0.923)
One-Child family (Ref. No)					
Yes	0.973	0.835	1.359	0.244	2.646(0.515–13.583)
Charitable medical assistance (Ref. No)					
Yes	−1.888	0.633	8.895	0.003^**^	0.151(0.044–0.524)

*CHE* Catastrophic Health Expenditure, *CNY* Chinese Yuan, *CI* confidence intervals

^*^P < 0.05, ^**^P < 0.01

## Discussion

This study found that families of children with leukemia experienced a heavy economic burden, with a high incidence of CHE. Following charitable medical assistance, the intervention group exhibited lower numerical values for OOP expenses and the incidence of CHE compared to the control group. Additionally, the study identified that the child’s age, family monthly income, and family size were also primary factors influencing the incidence of CHE.

According to Guangdong’s provincial government statistics, the per capita disposable income was CNY47,100 ($6,517) in 2022. The study showed that the OOP medical expenses for both of family groups were more than CNY200,000($27,632) after medical insurance reimbursement, approximately fivefold the per capita disposable income in Guangdong Province in 2022. This suggested that families of pediatric leukemia faced considerable economic risks, in accordance with studies conducted within a specialized tertiary hematology hospital [19]. The post-reimbursement CHE incidence for both of family groups was around 75%, significantly higher than the overall CHE incidence rate in China (27.4%) [20]. Consequently, without any financial assistance, families of children with leukemia were generally prone to experiencing CHE, a finding consistent with the research of Zhao [21]. The economic burden on families of children with leukemia may be attributed to several reasons. Firstly, the treatment costs for leukemia have been raised by the expenses of imported medicines, specialty drugs, advanced therapeutic techniques, and the use of disposable items to prevent infections [22]. Secondly, some leukemia treatments were not included in the medical insurance reimbursement coverage [23]. Thirdly, the majority of patients faced reduced medical reimbursements and higher living costs when seeking treatment away from home [24]. Fourthly, the majority of families, deprived of their primary income, must rely on their savings, loans, and charitable assistance to sustain basic medical treatment and daily life [25, 26].

After receiving charitable medical assistance, the intervention group demonstrated lower numerical values for OOP and the incidence of CHE, with the probability of experiencing CHE being just 0.151 times that of the control group. This indicated that charitable medical assistance served as a powerful supplement to basic medical insurance and serious illness insurance. The collaborative model with medical insurance reimbursement had played a positive supplementary role in reducing the financial burden on families of children with leukemia. It helped reduce the incidence of OOP expenses and CHE in families of pediatric leukemia, thereby playing a safety-net role. Research from the King Hussein Cancer Center/King Hussein Cancer Foundation and the Drug Recovery and Copay Assistance Program affirmed that charitable medical assistance was an essential financial support for families of children with leukemia [11, 27].

In addition to supplementary medical expense reimbursements, other comprehensive measures under the “Love Union Project” may also indirectly alleviate the financial burden faced by families of children with leukemia. Firstly, the pharmacoeconomic evaluation conducted for the project included two effective medications for treating pediatric leukemia within the scope of supplementary reimbursement. This measure has alleviated the financial burden of medical expenses for families of affected children to some extent [21]. Additionally, the project offers support such as compassionate transportation, temporary housing, and short-term employment opportunities for families seeking medical care away from home, potentially alleviating the economic burden associated with distant healthcare access [21]. Finally, the ward care support provided by social workers helps caregivers understand and prioritize infection prevention, potentially reducing healthcare expenditures [21].

This study found a positive correlation between monthly household income and the incidence of CHE in families of children with leukemia. Increased family income strengthened the ability to counteract the financial shock of major illnesses in children, improving the accessibility and affordability of medical services. Consequently, the likelihood of CHE was reduced, mirroring the results from two cross-national studies [28, 29]. This study also found that the risk of CHE for families with children aged 7–12 was 5.224 times higher than for those with children aged 0–6, consistent with the research by Huang et al. [30]. Children in this age group have limited self-care abilities but exhibit strong feelings of fear and resistance. In addition to closely monitoring the child’s conditions, caregivers must also provide increased support in treatment and dietary aspects. This additional care inevitably increases the likelihood of CHE for the family. Furthermore, the risk of CHE was higher in larger household-size families, possibly due to the caregiver’s challenges in looking after other family members [31]. Caregivers often need to focus all their attentions on taking care of their sick children, so they may need to send other family members who need care to a daycare center or rely on relatives for help [31]. This may result in additional financial costs, thereby exacerbating the family’s economic burden.

### Limitation

The limitations of this study were as follows. First, the cross-sectional design may introduce recall bias among respondents. Future studies should opt for large-sample, multicenter longitudinal research designs. Second, due to the online survey method and a significant refusal rate (13.3%), selection and measurement biases may exist. To mitigate these biases in questionnaire responses, professional training for enumerators was implemented. Third, although there was an imbalance in the distribution between the intervention and control groups during the treatment phase, subsequent subgroup and multivariable analyses indicated that this disparity wouldn’t significantly impact OOP or CHE for families of children with leukemia. Fourth, although the medical insurance reimbursement policies for the two groups were highly similar, minor differences did exist. Future research should further analyze the potential impacts of these differences. Finally, this study primarily examined the effects of the “Love Union Project” on supplementary medical reimbursement. Future research could further investigate the effectiveness of comprehensive measures such as caregiver support, material assistance, and support for cross-regional medical care.

## Conclusion

All in all, the high treatment costs severely strained the financial capacity of families of children with leukemia. After medical insurance reimbursement, families of children with leukemia still bore a certain degree of financial pressure. However, the medical assistance provided by the “Love Union Project” has mitigated CHE among the beneficiaries’ families. Future efforts should prioritize the role of charitable medical assistance as a safety net, encourage the active involvement of charitable organizations in supporting children with severe and major illnesses, and expedite the development of a multi-tiered medical security system.

## Supplementary Information

Supplementary Material 1.

## Data Availability

The datasets used and/or analysed during the current study are available from the corresponding author on reasonable request.

## Abbreviations

OOP: Out-of-pocket
CHE: Catastrophic health expenditure
CNY: Chinese Yuan
MIR: Medical Insurance Reimbursement
CI: Confidence Intervals
PPR: Post-Project Reimbursement
