# Supplementary material for: Does the charitable medical assistance program impact catastrophic medical expenditures for families of children with leukemia? An evidence-based study in China
Source: Int J Equity Health. 2025 Mar 17;24:75. doi: 10.1186/s12939-025-02442-1 (PMC11912703; doi:10.1186/s12939-025-02442-1)
Supplement: Supplementary file 1 — Supplementary Material 1. [file 12939_2025_2442_MOESM1_ESM.docx]

**Supplementary Materials**

**eTable 1** Demographic and clinical characteristics in two pediatric groups

**eTable 2** Comparison of OOP in families within the maintenance period across different scenarios

**eTable 3** Comparison of OOP in families within the consolidation period across different scenarios

**eTable 4** Comparison of CHE incidence in the subgroup within the maintenance period

**eTable 5** Comparison of CHE incidence in the subgroup within the consolidation period

| **eTable 1** **Demographic and** **clinical characteristics in two pediatric groups (N=121)** | | | | |
| --- | --- | --- | --- | --- |
| Variables | Intervention group  *N(%)* | Control group  *N(%)* | *χ^2^* | *P* |
| Sex |  |  | 0.017 | 0.898 |
| Male | 53(62.4) | 22(61.1) |  |  |
| Female | 32(37.7) | 14(38.9) |  |  |
| Age |  |  | 3.094 | 0.213 |
| 0-6 years | 9(10.6) | 8(22.2) |  |  |
| 7-12 years | 53(62.4) | 21(58.3) |  |  |
| 13-18 years | 23(27.1) | 7(19.4) |  |  |
| Disease type |  |  | 0.079 | 0.779 |
| ALL | 69(81.2) | 30(83.3) |  |  |
| Other disease types | 16(18.8) | 6(16.7) |  |  |
| Severity of illness |  |  | 0.814 | 0.666 |
| Low risk | 20(23.5) | 11(30.6) |  |  |
| Moderate risk | 42(49.4) | 15(41.7) |  |  |
| High risk | 23(27.1) | 10(27.8) |  |  |
| Treatment stage |  |  | 11.156 | 0.001^**^ |
| Maintenance period | 38(44.7) | 28(77.8) |  |  |
| Consolidation period | 47(55.3) | 8(22.2) |  |  |
| Transplant status |  |  | 1.296 | 0.255 |
| Yes | 33(38.8) | 18(50.0) |  |  |
| No | 52(61.2) | 18(50.0) |  |  |
| Infection status |  |  | 2.723 | 0.099 |
| Yes | 47(55.3) | 14(38.9) |  |  |
| No | 38(44.7) | 22(61.1) |  |  |
| Comorbidities |  |  | 3.391 | 0.066 |
| Yes | 12(14.1) | 1(2.8) |  |  |
| No | 73(85.9) | 35(97.2) |  |  |
| Time since diagnosis |  |  | 0.213 | 0.644 |
| 0-24 Months | 18(21.2) | 9(25.0) |  |  |
| >24 Months | 67(78.8) | 27(75.0) |  |  |
| Frequency of hospital visits |  |  | 2.325 | 0.344 |
| 1-2 times monthly | 60(70.6) | 21(58.3) |  |  |
| 3-4 times monthly | 9(10.6) | 7(19.4) |  |  |
| ≥5 times monthly | 16(18.8) | 8(22.2) |  |  |
| Household size |  |  | <0.001 | 0.997 |
| 1-4 members | 26(30.6) | 11(30.6) |  |  |
| 5 or more | 59(69.4) | 25(69.4) |  |  |
| One-Child family |  |  | 2.724 | 0.099 |
| Yes | 9(10.6) | 0(0.0) |  |  |
| No | 76(89.4) | 36(100.0) |  |  |
| Monthly family income |  |  | 0.646 | 0.886 |
| [0~4000] CNY | 21(24.7) | 11(30.6) |  |  |
| (4000~5500] CNY | 20(23.5) | 9(25.0) |  |  |
| (5500~8000] CNY | 21(24.7) | 8(22.2) |  |  |
| (8000~+∞) CNY | 23(27.1) | 8(22.2) |  |  |

Note: ALL, Acute Lymphocytic Leukemia; CNY, Chinese Yuan; ^*^*P*<0.05, ^**^*P*<0.01, ^***^*P*<0.001.

| **eTable 2** **Comparison of OOP in families within the maintenance** **period across different scenarios** | | | | | | |
| --- | --- | --- | --- | --- | --- | --- |
| Scenarios | Intervention group(n=38) | | Control group(n=28) | | *Z* | *P* |
|  | Median | Reduction (%) | Median | Reduction (%) |  |  |
| Pre-MIR | CNY418,000 | / | CNY447,500 | / | -0.500 | 0.617 |
| Post-MIR | CNY220,000 | 47.4 | CNY217,200 | 51.5 | -0.260 | 0.795 |
| PPR | CNY190,000 | 13.6 | / | / | -0.376 | 0.707 |

Note: OOP, Out-of-Pocket; MIR, Medical Insurance Reimbursement; CNY, Chinese Yuan; PPR, Post-Project reimbursement, the scenario was conducted on the intervention group with the project aid and compared with the control group after medical insurance reimbursement.

| **eTable 3 Comparison of OOP in families within the consolidation period across different scenarios** | | | | | | |
| --- | --- | --- | --- | --- | --- | --- |
| Scenarios | Intervention group(n=47) | | Control group(n=8) | | *Z* | *P* |
|  | Median | Reduction (%) | Median | Reduction (%) |  |  |
| Pre-MIR | CNY430,000 | / | CNY354,500 | / | -1.146 | 0.262 |
| Post-MIR | CNY250,000 | 41.9 | CNY210,000 | 40.8 | -0.967 | 0.340 |
| PPR | CNY203,000 | 18.8 | / | / | -0.143 | 0.898 |

Note: OOP, Out-of-Pocket; MIR, Medical Insurance Reimbursement; CNY, Chinese Yuan; PPR, Post-Project reimbursement, the scenario was conducted on the intervention group following project aid and compared with the control group after medical insurance reimbursement.

| **eTable 4** **Comparison of CHE incidence in the subgroup within the maintenance** **period** | | | | | | |
| --- | --- | --- | --- | --- | --- | --- |
| Scenarios | Intervention group(n=38) | | Control group(n=28) | | *χ^2^* | *P* |
|  | CHE, n (%) | Reduction (%) | CHE, n (%) | Reduction (%) |  |  |
| Pre-MIR | 32(84.2) | / | 26(92.9) | / | 0.465 | 0.495 |
| Post-MIR | 28(73.7) | 12.5 | 22(78.6) | 15.4 | 0.210 | 0.647 |
| PPR | 24(63.2) | 14.3 | / | / | 1.813 | 0.178 |

Note: CHE, Catastrophic Health Expenditure; MIR, Medical Insurance Reimbursement; PPR, Post-Project reimbursement, the scenario was conducted on the intervention group with the project aid and compared with the control group after medical insurance reimbursement.

| **eTable 5** **Comparison of CHE incidence in the subgroup within the consolidation period** | | | | | | |
| --- | --- | --- | --- | --- | --- | --- |
| Scenarios | Intervention group(n=47) | | Control group(n=8) | | *χ^2^* | *P* |
|  | CHE, n (%) | Reduction (%) | CHE, n (%) | Reduction (%) |  |  |
| Pre-MIR | 46(89.4) | / | 5(62.5) | / | 2.102 | 0.147 |
| Post-MIR | 36(76.6) | 14.3 | 5(62.5) | 0 | 0.166 | 0.684 |
| PPR | 32(68.1) | 11.1 | / | / | ＜0.001 | 1.000 |

Note: CHE, Catastrophic Health Expenditure; MIR, Medical Insurance Reimbursement; PPR, Post-Project reimbursement, the scenario was conducted on the intervention group with the project aid and compared with the control group after medical insurance reimbursement.
